# Induction of Broad-Spectrum Protective Immunity against Disparate *Cryptococcus* Serotypes

**DOI:** 10.3389/fimmu.2017.01359

**Published:** 2017-10-30

**Authors:** Marley C. Caballero Van Dyke, Ashok K. Chaturvedi, Sarah E. Hardison, Chrissy M. Leopold Wager, Natalia Castro-Lopez, Camaron R. Hole, Karen L. Wozniak, Floyd L. Wormley

**Affiliations:** ^1^Department of Biology, The University of Texas at San Antonio, San Antonio, TX, United States; ^2^The South Texas Center for Emerging Infectious Diseases, The University of Texas at San Antonio, San Antonio, TX, United States

**Keywords:** *Cryptococcus neoformans*, *Cryptococcus gattii*, cryptococcosis, host–fungal interaction, fungal vaccines, fungal immunology

## Abstract

Cryptococcosis is a fungal disease caused by multiple *Cryptococcus* serotypes; particularly *C. neoformans* (serotypes A and D) and *C. gattii* (serotypes B and C). To date, there is no clinically available vaccine to prevent cryptococcosis. Mice given an experimental pulmonary vaccination with a *C. neoformans* serotype A strain engineered to produce interferon-γ, denoted H99γ, are protected against a subsequent otherwise lethal experimental infection with *C. neoformans* serotype A. Thus, we determined the efficacy of immunization with *C. neoformans* strain H99γ to elicit broad-spectrum protection in BALB/c mice against multiple disparate *Cryptococcus* serotypes. We observed significantly increased survival rates and significantly decreased pulmonary fungal burden in H99γ immunized mice challenged with *Cryptococcus* serotypes A, B, or D compared to heat-killed H99γ (HKH99γ) immunized mice. Results indicated that prolonged protection against *Cryptococcus* serotypes B or D in H99γ immunized mice was CD4^+^ T cell dependent and associated with the induction of predominantly Th1-type cytokine responses. Interestingly, immunization with H99γ did not elicit greater protection against challenge with the *Cryptococcus* serotype C tested either due to low overall virulence of this strain or enhanced capacity of this strain to evade host immunity. Altogether, these studies provide “proof-of-concept” for the development of a cryptococcal vaccine that provides cross-protection against multiple disparate serotypes of *Cryptococcus*.

## Introduction

Cryptococcosis is a worldwide fungal disease caused by species in the *Cryptococcus neoformans*/*Cryptococcus gattii* species complex (1). *C. neoformans* and *C. gattii* cause pneumonia in immunocompromized and immunocompetent individuals and can disseminate to the central nervous system resulting in life-threatening meningoencephalitis. Cryptococcal meningoencephalitis is the most common disseminated fungal disease in AIDS patients (2) and is responsible for 15% of AIDS-related deaths (3). The former species *C. neoformans* is divided into two species, *C. neoformans* and *C. deneoformans*. *C. neoformans* (serotype A) is distributed worldwide with the highest amount of disease cases occurring in sub-Saharan Africa where approximately 21.7 million people currently live with AIDS, and *C. deneoformans* (serotype D) is predominantly geographically restricted to Europe and Latin America (3–5). Conversely, *C. gattii* (serotype B *C. deuterogattii* and serotype C *C. bacillisporus*) predominantly occurs in tropical and subtropical climates (6–8). However, cryptococcosis due to *C. gattii* is also observed in more temperate climates including British Columbia, Canada, and southwest, southeast, northwest, and northeast regions of the USA and in Mediterranean Europe (9–16). Species in the *C. neoformans*/*C. gattii* complex can cause disease in both apparently healthy individuals and immunocompromized hosts such as AIDS patients, individuals on prolonged treatment with corticosteroids, and in patients on immunosuppressive drugs to prevent rejection of solid organ transplants [reviewed by Kwon-Chung et al. (17)] (7, 8, 18–20). There is a higher occurrence of *C. gattii* disease in immunocompetent individuals compared to *C. neoformans* (20–22). Currently, there is no vaccine clinically available to prevent cryptococcosis and current drug therapies are often rendered ineffective due to the development of drug resistance by *Cryptococcus* or drug toxicity (23). Thus, the overall incidence of disease and mortality associated with cryptococcosis and the potential expanded geographic distribution of the pathogen indicates an urgent need for immunotherapies and/or vaccines to combat cryptococcosis.

Results from multiple studies in both humans and animal models suggest that cell-mediated immunity (CMI) by Th1-type CD4^+^ T cells is the primary host defense against cryptococcosis (24, 25). However, studies suggest that the host immune response against *C. gattii* and *C. neoformans* differ in that *C. gattii* infection appears to be more immune suppressive, which may explain the disparate clinical presentation displayed by these different species (26–31). Differences in the host response to various *Cryptococcus* serotypes complicate efforts to devise a vaccine that can provide broad-spectrum protection against cryptococcosis caused by disparate *Cryptococcus* serotypes.

Historically, several approaches have been employed to develop an anti-*Cryptococcus* vaccine that generates protective antibody mediated and/or cell-mediated immune responses against *Cryptococcus* [reviewed in Ref. (32).]. Recently, studies have demonstrated that mice given an immunization with glucan particles (GPs) packaged with alkaline extracts from mutant *C. neoformans* or *C. gattii* strains develop increased survival against cryptococcosis (33). Other, studies showed cross-protection of mice immunized with a heat-killed chitosan deficient *C. neoformans* strain against challenge with *C. neoformans* or *C. gattii* (34). Rella and colleagues showed that mice vaccinated with a *C. neoformans* mutant strain that lacks the enzyme sterylglucosidase (Δ*sgl*1) are protected against challenge with both *C. neoformans* H99 and *C. gattii* R265 strains (35).

Studies have shown that mice immunized with a *C. neoformans* strain H99, serotype A, engineered to produce murine interferon (IFN)-γ, denoted H99γ, develop protective Th1-type immune responses against a subsequent otherwise lethal pulmonary challenge with wild-type *C. neoformans* strain H99 (25, 36–40). The presence of CD4^+^ and/or CD8^+^ T cells are required for protection during the immunization phase with H99γ but not during subsequent challenge with *C. neoformans* strain H99 (40). These results and those by Rella et al. highlight that protection against pulmonary cryptococcosis in immunocompetent hosts can be maintained in immunosuppressed hosts and that the development of a prophylactic vaccine against cryptococcosis is feasible. Nonetheless, no studies have shown that protection elicited following immunization with one *Cryptococcus* serotype provides similar levels of cross-protection against all four *Cryptococcus* serotypes.

Consequently, the objective of these studies was to determine the potential for an anti-cryptococcal vaccine to provide broad-spectrum protection against multiple disparate *Cryptococcus* serotypes. We therefore determined the efficacy of immunization with *C. neoformans* strain H99γ to elicit protection against experimental pulmonary challenge with disparate cryptococcal serotypes in mice. We demonstrate that immunization with *C. neoformans* strain H99γ, serotype A, elicits a predominantly protective Th1-type immune response against challenge with different serotypes of *Cryptococcus*. These data support the premise that development of a broad-spectrum prophylactic vaccine against cryptococcosis is achievable.

## Materials and Methods

### Mice

Female BALB/c (National Cancer Institute/Charles River Laboratories) 4–6 weeks of age were used throughout these studies. Mice were housed at the University of Texas at San Antonio Small Animal Laboratory Vivarium and all animal experiments were conducted following NIH guidelines for housing and care of laboratory animals and in accordance with protocols approved by the Institutional Animal Care and Use Committee (protocol number MU021) of the University of Texas at San Antonio.

### Strains and Media

*Cryptococcus neoformans* strains H99 (serotype A, mating type α), *C. deuterogattii* strain R265 (serotype B; a kind gift from Dr. Joseph Heitman of Duke University Medical Center in Durham, NC, USA), *C. bacillisporus* strain WSA87 (serotype C), and *C. deneoformans* strain R4247 (serotype D) (each kind gifts from Dr. Brian Wickes of the UT Health San Antonio, San Antonio, TX, USA), and H99γ [an IFN-gamma producing *C. neoformans* strain derived from H99 (38)] were recovered from 15% glycerol stocks stored at −80°C prior to use in the experiments described herein. The strains were maintained on yeast-extract-peptone-dextrose (YPD) media (1% yeast extract, 2% peptone, 2% dextrose, and 2% Bacto agar). Yeast cells were grown for 16 h at 30°C with shaking in YPD broth, collected by centrifugation, washed three times with sterile PBS, and viable yeasts were quantified using trypan blue dye exclusion on a hemacytometer.

### Pulmonary Cryptococcal Infections

Cryptococcal infections were initiated as previously described (25, 41). Briefly, mice were anesthetized with 2% isoflurane using a rodent anesthesia device (Eagle Eye Anesthesia, Jacksonville, FL, USA) and given an intranasal inoculation with 1 × 10^4^ CFU of *Cryptococcus* strains H99, R265, WSA87, or R4247 in 50 µl of sterile PBS. Alternatively, mice were given an intranasal immunization with 1 × 10^4^ CFU of *C. neoformans* strain H99γ or heat-killed H99γ (HKH99γ) yeasts in 50 µl of sterile PBS, allowed 70 days to resolve the infection and subsequently given an intranasal challenge with 1 × 10^4^ CFU of H99, R265, WSA87, or R4247 in 50 µl of sterile PBS. The inocula used for immunizations and challenges were verified by quantitative culture on YPD agar. The mice were monitored by inspection twice daily. Mice were euthanized on pre-determined days post challenge and lung tissues excised using aseptic technique. Tissues were homogenized in 1 ml of sterile PBS followed by culture of 10-fold dilutions of each tissue on YPD agar supplemented with chloramphenicol (Mediatech Inc., Herndon, VA, USA). CFU were enumerated following incubation at 30°C for 48 h. Alternatively, mice intended for survival analysis were monitored by inspection twice daily and euthanized if they appeared to be in pain or moribund (weight loss, ataxia, listlessness, or failure to groom). Mice were euthanized using CO_2_ inhalation followed by cervical dislocation.

### T Cell Depletion

Mice were immunized with 1 × 10^4^ CFU of *C. neoformans* strain H99γ as described above. After 70 days of rest, mice were depleted of CD4^+^ T cells, CD8^+^ T cells, or both CD4^+^/CD8^+^ T cell subsets *via* intraperitoneal administration of anti-CD4 (clone GK1.5) and anti-CD8 (clone 2.43) antibodies (Table 1) (each from Cell Culture Company/NCCC, Minneapolis, MN, USA) or given isotype control antibody (rat IgG_2b_) (BioXCell, Lebanon, NH, USA). Each mouse received 200 µg of GK1.5 and/or 2.43 or control rat IgG_2b_ antibodies in a volume of 200 µl PBS 48 h prior to challenge with H99, R265, WSA87, or R4247 and weekly thereafter during the observation period. The efficiency of T cell depletion in lungs and spleens was assessed by flow cytometric analysis using anti-CD4 and anti-CD8 antibodies that bind epitopes of the CD4 and CD8 protein at locations distinct from GK1.5 and 2.43. Efficiency was determined to be >98% at each anatomic location for each depletion via comparison of T cell subsets in treated mice with those in control animals.

**Table 1:** Antibodies used in flow cytometry.

mAb	Clone	Manufacturer
CD45R (B220)-PE	RA3-6B2	BD Biosciences
CD11c-Pe-Cy7	N418	eBiosciences
CD11c-PE	N418	eBiosciences
CD11b-Pe-Cy7	M1170	eBiosciences
CD45-APC	30-F11	eBiosciences
CD45-Pe-Cy7	30-F11	eBiosciences
F4/80-APC	BM8	Life Technologies
Ly6G-PE	1A8	BD Biosciences
T cell mix-CD8a APC, CD3e, Pe-Cy7, CD4 PE	CD8a 53-6.7, CD3e-145-2C11, CD4 RM4-5	BD Biosciences
CD19-PE	1D3	BD Biosciences
Siglec-F-PE	E50-2440	BD Biosciences
CD3e-Pe-Cy7	145-2C11	eBiosciences
CD4-PE	RM4-5	BD Biosciences
Intracellular flow		
Interferon-γ-APC	XMG1.2	eBiosciences
IL-4-APC	11B11	eBiosciences
IL-17A-APC	eBio17B7	eBiosciences
Depletion antibodies		
Anti-CD4	GK1.5	Cell Culture Company
Anti-CD8	2.43	Cell Culture Company

### Cytokine Analysis

As previously described (25, 41), cytokine levels in lung tissues were analyzed using the Bio-Plex protein array system (Luminex-based technology; Bio-Rad Laboratories, Hercules, CA, USA). Briefly, the left lobe of the lung was excised and homogenized in ice-cold sterile PBS (1 ml). An aliquot (50 µl) was taken to quantify the pulmonary fungal burden and an anti-protease buffer solution containing PBS, protease inhibitors (inhibiting cysteine, serine, and other metalloproteinases), and 0.05% Triton X-100 was added to the homogenate and then clarified by centrifugation (800 × *g*) for 10 min. Supernatants from pulmonary homogenates were assayed for the presence of IL-1α, IL-1β, IL-1, IL-3, IL-4, IL-5, IL-6, IL-9, IL-10, IL-12(p40), IL-12(p70), IL-13, IL-17A, CCL5/RANTES, CCL11/Eotaxin, CXCL1/KC, CCL3/MIP-1α, CCL4/MIP-1β, CCL2/MCP-1, G-CSF, GM-CSF, TNF-α, and IFN-γ.

### Pulmonary Leukocyte Isolation

Lungs were excised on days 3, 7, and 14 post challenge and digested enzymatically at 37°C for 30 min in 10 ml of digestion buffer (RPMI 1640 and 1 mg/ml of collagenase type IV) (Sigma Chemical Co., St. Louis, MO, USA) with intermittent (every 10 min) stomacher homogenizations as previously described (25, 41). The digested tissues were then filtered through sterile nylon filters of various pore sizes (70 and 40 µm; BD Biosciences) and washed with sterile HBSS to enrich for leukocytes. Erythrocytes were lysed by incubation in NH_4_Cl buffer (0.859% NH_4_Cl, 0.1% KHCO_3_, 0.0372% Na_2_EDTA pH 7.4) for 3 min on ice followed by the addition of a twofold excess of PBS. The leukocyte population was then washed twice with sterile PBS, suspended in sterile PBS + 2% heat-inactivated fetal bovine serum (FACS buffer), and enumerated in a hemacytometer using trypan blue dye exclusion. Flow cytometry analysis was used to determine the percentage of each leukocyte population as well as the absolute number of total leukocytes (CD45^+^) within the lung cell suspension for standardization of hemacytometer counts.

### Flow Cytometry

Standard methodology was employed for the direct immunofluorescence of pulmonary leukocytes (25, 41). Briefly, in 96-well U-bottom plates, 100 µl containing 1 × 10^6^ cells in PBS plus 2% FBS (FACS buffer) were incubated with Fc block (BD Biosciences) diluted in FACS buffer for 5 min to block nonspecific binding of antibodies to cellular Fc receptors. An optimal concentration of fluorochrome-conjugated antibodies (0.125—1 µg/1 × 10^6^ cells) as listed in Table 1 was added in various combinations to allow for dual or triple staining, and cells were then incubated for 30 min at 4°C. Cells were washed three times with FACS buffer and fixed in 200 µl of 2% ultrapure formaldehyde (Polysciences) diluted in FACS buffer (fixation buffer). Cells analyzed for intracellular cytokine staining were fixed in fixation buffer for 10 min in the dark at room temp. The cells were then permeabilized with 0.1% saponin (diluted in FACS buffer) and incubated for 10 min in the dark at room temperature. Antibodies listed in Table 1 were subsequently added at optimal concentrations, and cells incubated at 4°C for 30 min. Following incubation, cells were washed three times with 0.1% saponin buffer and resuspended in fixation buffer. Cells incubated with either FACS buffer alone or single fluorochrome-conjugated Abs were used to determine positive staining and spillover/compensation calculations, and background fluorescence determined with FlowJo Software (FlowJo, LLC, Ashland, OR, USA). Raw data were collected with the BD FACSArray flow cytometer (BD Biosciences) and then analyzed using FlowJo Software. Dead cells were excluded on the basis of forward angle and 90° light scatter. For data analyses, 30,000 events (cells) were evaluated from a predominantly leukocyte population identified by back gating from CD45^+^ stained cells. The absolute number of total leukocytes was quantified by multiplying the total number of cells observed by hemacytometer counting by the percentage of CD45^+^ cells determined by flow cytometry. The absolute number of leukocytes (CD45^+^ cells), T cells (CD4^+^/CD3^+^ and CD8^+^/CD3^+^), CD19^+^/CD45^+^, Ly6G^+^/CD45^+^, F4/80^+^/CD45^+^, CD11b^int^/CD11c^+^/CD45^+^, CD11b^hi^CD11c^+^/CD45^+^, and Siglec-F^+^/CD11b^+^ was determined by multiplying the percentage of each gated population by the total number of CD45^+^ cells. Gating strategies are provided as supplemental materials. Intracellular cytokine staining is represented in histograms with unstained cells as a control.

### Statistical Analysis

The unpaired Student’s *t* test (two-tailed) was used to analyze the immunization studies fungal burden, pulmonary cell populations, and cytokine/chemokine data where appropriate using GraphPad Prism version 5.00 for Windows (GraphPad Prism Software, San Diego, CA, USA). Survival data were analyzed using the log-rank test (GraphPad Software). Significant differences were defined as *p < 0.05, **p < 0.005, or ***p < 0.0001.

## Results

### Immunization with *C. neoformans* Strain H99γ Elicits Protection against Disparate *Cryptococcus* Serotypes

**Figure 1:**
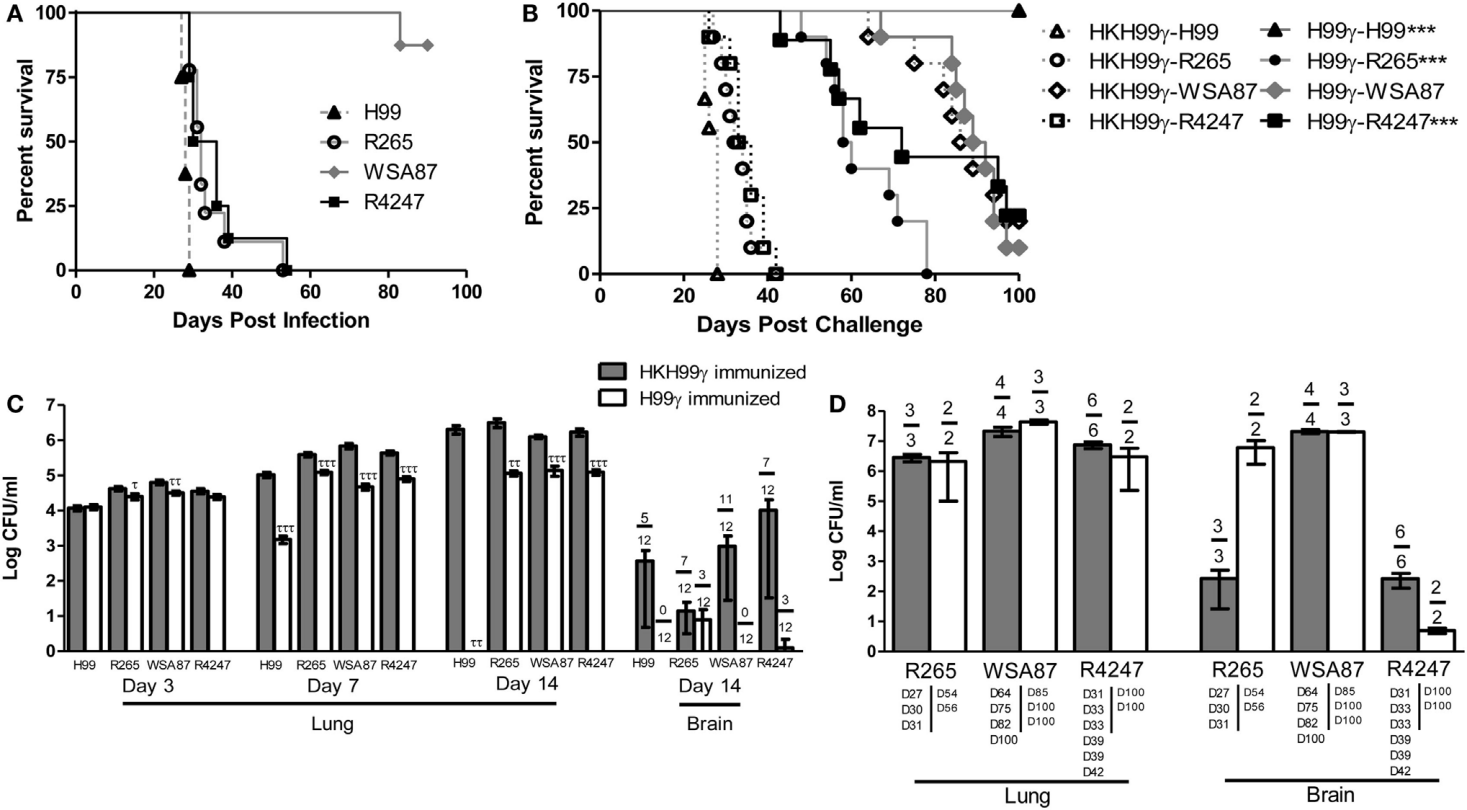
Virulence of disparate *Cryptococcus* serotypes in non-immunized or serotype A immunized mice. BALB/c mice received an intranasal inoculation **(A)** with 1 × 10^4^ CFU of *Cryptococcus* strain H99 (serotype A), R265 (serotype B), WSA87 (serotype C), or R4247 (serotype D). Mice were observed for up to 90 days for mortality analysis. Alternatively, BALB/c mice received an intranasal immunization **(B)** with 1 × 10^4^ CFU of either H99γ or HKH99γ, allowed 70 days to resolve the infection, and subsequently challenged with individual serotypes of *Cryptococcus*. Mice were observed for up to 100 days post challenge for survival analysis **(B)** or, alternatively pulmonary and brain fungal burden determined at days 3, 7, and 14 post challenge **(C)**. Pulmonary and brain fungal burden were analyzed from mice that appeared moribund or sacrificed at 100 days post challenge with the day the mice were sacrificed indicated below the bars **(D)**. Survival data shown are from one experiment, using 8–10 mice per group. Fungal burden data are cumulative of three experiments using 4 mice per group per time point. Numbers above the bars represent the number of mice positive for *Cryptococcus*. *p < 0.05, **p < 0.005, ***p < 0.0001. *Significant increase compared to HKH99γ; ^τ^*p* < 0.05, ^ττ^*p* < 0.005, ^τττ^*p* < 0.0001. ^τ^Significant decrease compared to HKH99γ.

Previous studies in our lab demonstrated that experimental pulmonary infection with *C. neoformans* strain H99γ in mice results in clearance of the acute infection and induction of protective immunity against a subsequent otherwise lethal challenge with a WT *C. neoformans* strain H99 that does not produce IFN-γ (25, 36–40). Nevertheless, studies to date have not demonstrated that protective immunity generated against one serotype of *Cryptococcus* affords protection against multiple disparate *Cryptococcus* serotypes. Consequently, we sought to determine the level of cross-protection that is generated in mice immunized with *C. neoformans* strain H99γ, a serotype A strain, against pulmonary infection with disparate serotypes of *Cryptococcus*. To establish a baseline prior to evaluating if immunization with *Cryptococcus* strain H99γ, serotype A, enhanced protection against challenge with disparate *Cryptococcus* serotypes, we first determined the relative virulence of each serotype in non-immunized mice. To do this, BALB/c mice were given an experimental pulmonary infection with either *Cryptococcus* strain H99 (serotype A), R265 (serotype B), WSA87 (serotype C), or R4247 (serotype D) and evaluated for mortality (Figure 1A). As shown in Figure 1A, experimental pulmonary infection with *Cryptococcus* strains H99, R265, and R4247 resulted in 100% mortality with median survival times of 28, 32, and 33 days, respectively. In contrast, we observed 87.5% survival in mice given an experimental pulmonary infection with WSA87. *Cryptococcus* strain WSA87, serotype C, appeared to be significantly less virulent compared to all the other strains tested during our analysis.

Next, we tested the potential of mice immunized with *C. neoformans* strain H99γ (serotype A) to be protected against challenge with *Cryptococcus* serotypes B, C, or D. For this, mice were immunized with *C. neoformans* strain H99γ or heat-killed H99γ (HKH99γ), rested for 70 days, and separate groups subsequently given an experimental pulmonary challenge with each *Cryptococcus* serotype. We observed a 100% survival rate in mice immunized with H99γ and challenged with H99, while the HKH99γ immunized mice challenged with H99 demonstrated a median survival time of 28 days post challenge (Figure 1B), confirming our previous observations (38, 40). Mice immunized with H99γ and then challenged with either R265 (*C. gattii* serotype B) or R4247 (*C. deneoformans* serotype D) exhibited median survival times of 59 and 72 days post challenge, respectively, and significantly increased survival rates compared to HKH99γ immunized mice challenged with the same strain (median survival of 33 and 34.5 days in R265 and R4247 challenged mice, respectively; Figure 1B). Additionally, we observed a 20% survival rate in protectively immunized mice challenged with R4247 at 100 days post challenge. Figure 1B shows that no significant difference in mortality was observed in H99γ immunized mice challenged with WSA87 (median survival time of 90.5 days) compared to HKH99γ immunized mice challenged with the same strain (median survival time of 87.5 days). The mortality of mice immunized with HKH99γ and challenged with each *Cryptococcus* serotype mirrored the mortality rates of non-immunized mice given an experimental infection with each serotype and provided no additional protection (Figures 1A,B).

Pulmonary fungal burden was also quantified at days 3, 7, and 14 post challenge in H99γ or HKH99γ immunized mice challenged with each individual serotype (Figure 1C). Mice immunized with HKH99γ and challenged with each species showed progressive growth of each serotype in the lungs. In contrast, we observed significantly less pulmonary fungal burden as early as day 3 in H99γ immunized mice challenged with R265 and WSA87 compared to HKH99γ immunized mice (Figure 1C). Additionally, H99γ immunized mice showed significantly less pulmonary fungal burden following challenge with all serotypes on days 7 and 14 post challenge compared to HKH99γ immunized mice challenged with the same strain. Brain fungal burden was also quantified at day 14 post challenge and a trend (although not statistically significant) toward a reduction of brain fungal burden together with the number of mice negative for *Cryptococcus* in the brain was observed in H99γ immunized mice challenged with each serotype compared to their HKH99γ immunized counterparts (Figure 1C). Overall, our data show that mice immunized with H99γ, serotype A, display significant protection against challenge with serotypes A, B, or D of *Cryptococcus*.

Figure 1D demonstrates the fungal burden of moribund mice or mice sacrificed at the conclusion of the study. The HKH99γ immunized mice challenged with R265 appeared to have similar pulmonary fungal burden and less brain fungal burden at time of death compared to H99γ immunized mice challenged with R265 (Figure 1D). No significant differences in pulmonary or brain fungal burden were observed between H99γ and HKH99γ immunized mice challenged with WSA87 (Figure 1D). Both groups of mice immunized with HKH99γ or H99γ and challenged with R4247 appear to have similar pulmonary fungal burden and lower brain fungal burden at sacrifice with the H99γ immunized mice surviving longer. However, we cannot definitively state if the mice succumbed to pulmonary and/or brain fungal burden. Overall, immunization with H99γ appeared to prolong the survival of mice challenged with each serotype.

### Pulmonary Leukocyte Recruitment during Pulmonary Cryptococcosis Is Increased in H99γ Immunized Mice Challenged with Each *Cryptococcus* Serotype

**Figure 2:**
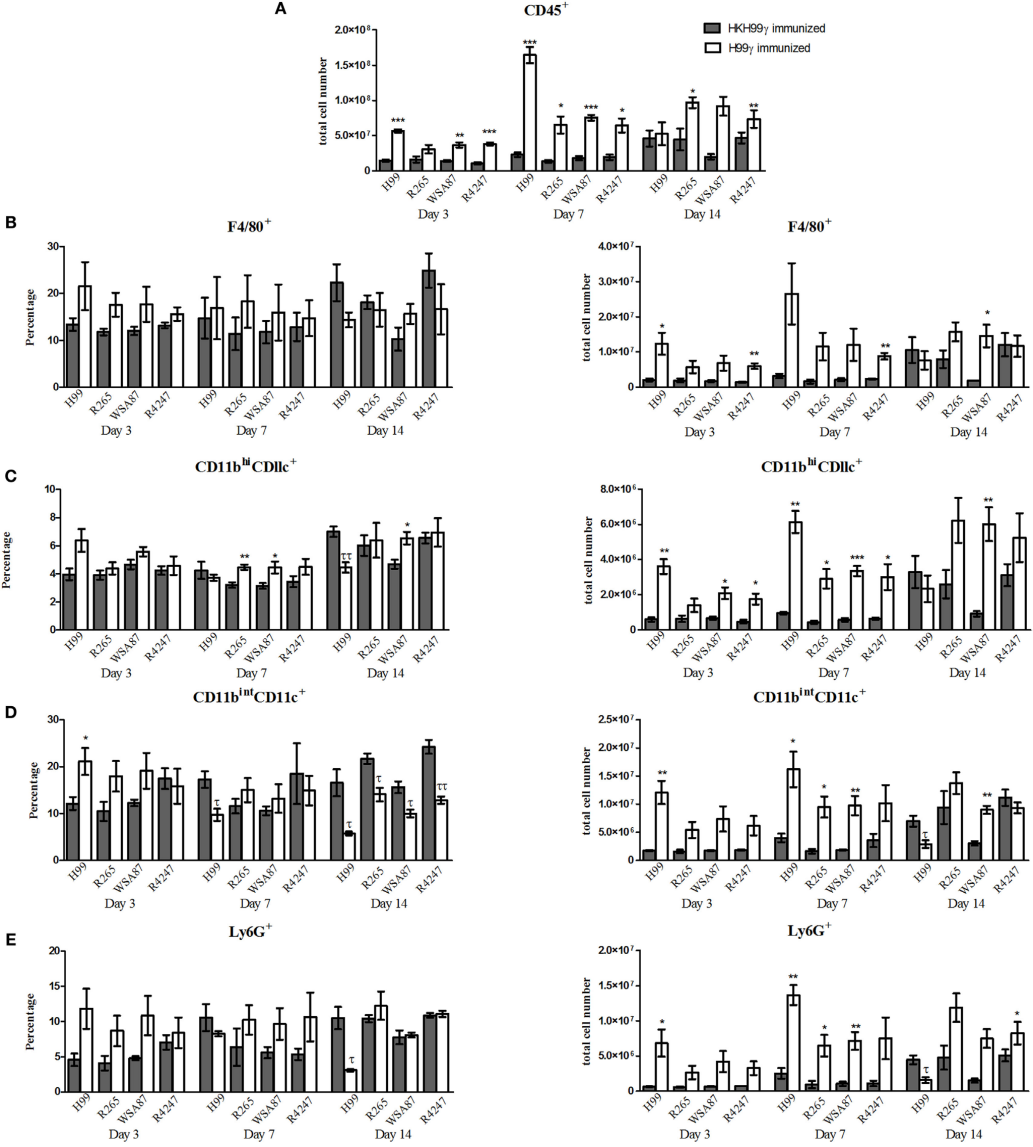

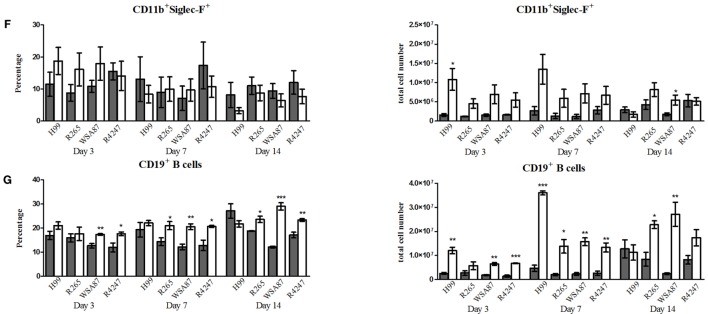
Pulmonary leukocyte populations in immunized mice challenged with different serotypes of *Cryptococcus*. BALB/c mice were immunized intranasally with 1 × 10^4^ CFU of either H99γ or HKH99γ, allowed 70 days to resolve the infection, and subsequently challenged with individual serotypes of *Cryptococcus*. At days 3, 7, and 14 post challenge, lungs were excised, tissues digested, and pulmonary infiltrates were analyzed by flow cytometry. Leukocytes were labeled with anti-CD45 **(A)** or dual labeled with anti-CD45 and antibodies for specific cell types **(B–G)** and analyzed by flow cytometry. Data shown are the mean ± SEM of absolute cell numbers or percentage from three independent experiments performed using 4 mice per group per experiment. *p < 0.05, **p < 0.005, ***p < 0.0001. *Significant increase compared to HKH99γ; ^τ^*p* < 0.05, ^ττ^*p* < 0.005, ^τττ^*p* < 0.0001. ^τ^Significant decrease compared to HKH99γ.

Our next goal was to examine the pulmonary inflammatory response in immunized mice during infection with the various representative *Cryptococcus* serotypes. We observed a significant increase in the total number of leukocytes recruited to the lungs of H99γ immunized mice challenged with H99 at days 3 and 7 post challenge compared to HKH99γ immunized mice (Figure 2A). Additionally, we observed significant increases in leukocyte recruitment to the lungs of H99γ immunized mice challenged with R265 on days 7 and 14 post challenge, H99γ immunized mice challenged with WSA87 on days 3 and 7 post challenge, and H99γ immunized mice challenged with R4247 at each time point post challenge compared to their HKH99γ immunized counterparts (Figure 2A). We observed an increased total cell number of F4/80^+^ cells in the lungs of H99γ immunized mice challenged with H99 at day 3, R4247 at days 3 and 7, and WSA87 at day 14 post challenge compared to HKH99γ immunized mice (Figure 2B). We observed a significant increase in the total number of CD11b^hi^/CD11c^+^ cells recruited to the lungs of H99γ immunized mice challenged with H99, WSA87, or R4247 at day 3, each serotype at day 7 post challenge, and in H99γ immunized mice challenged with WSA87 at day 14 post challenge compared to HKH99γ immunized mice challenged with the same serotype (Figure 2C). Figure 2C also shows a significant increase in the percentage of CD11b^hi^/CD11c^+^ cells in H99γ immunized mice challenged with R265 or WSA87 at day 7, and WSA87 at day 14 post challenge. There was also a significant increase in the total number of CD11b^int^CD11c^+^ cells in H99γ immunized mice challenged with H99 at days 3 and 7, R265 at day 7 post challenge, and WSA87 at days 7 and 14 post challenge (Figure 2D). Figure 2D also shows a significant increase in CD11b^int^CD11c^+^ cells in H99γ immunized mice challenged with H99 at day 3, while we observe a significant decrease in these cells at day 7 in H99 challenged mice, and across each serotype at day 14 post challenge. The recruitment of Ly6G^+^ cells to the lungs was significantly higher in H99γ immunized mice challenged with H99 at days 3 and 7 post challenge, R265 or WSA87 at day 7 post challenge, and in H99γ immunized mice challenged with R4247 on day 14 post challenge compared to their HKH99γ immunized counterparts (Figure 2E). While mice immunized with H99γ and challenged with H99 are clearing the infection, these mice show a significant decrease in the total number and percentage of Ly6G^+^ cells at day 14 post infection compared to HKH99γ immunized mice (Figure 2E). We observed a significant increase in the total number of Siglec-F^+^ cells in mice immunized with H99γ and challenged with H99 at day 3 post challenge and WSA87 challenged mice at day 14 post challenge (Figure 2F). We observed a significant increase in the total number of B cells in mice immunized with H99γ and challenged with H99, WSA87, or R4247 at day 3 post challenge, across each serotype at day 7 post challenge, and R265 or WSA87 at day 14 post challenge (Figure 2G). For the percentage of B cells, we observed a significant increase in H99γ immunized mice challenged with WSA87 or R4247 across each time point and in mice challenged with R265 at days 7 and 14 post challenge (Figure 2G). Overall, our data show significantly increased pulmonary recruitment of F4/80^+^ cells, CD11b^+^/CD11c^+^ cells, B cells, and Ly6G^+^ cells in mice immunized with the serotype A *Cryptococcus* strain H99γ following challenge with various other *Cryptococcus* serotypes which, altogether, may contribute to the increased protective responses observed in these mice.

### Immunization with H99γ Elicits Elevated Proinflammatory and a Predominant Th1-Type Cytokine Response following Challenge with Disparate *Cryptococcus* Serotypes

The pulmonary fungal burden and mortality analysis suggested that mice immunized with H99γ were able to mount protective responses against challenge with disparate *Cryptococcus* serotypes. Therefore, we evaluated cytokine levels in the lungs of H99γ or HKH99γ immunized mice following challenge with each *Cryptococcus* serotype. Lung homogenates were prepared from pulmonary tissues of mice on days 3, 7, and 14 post challenge with *Cryptococcus* serotypes A, B, C, or D, and the levels of Th1 associated (IFN-γ, IL-2, IL-12p70), Th2 associated (IL-4 and IL-5), immunoregulatory (IL-10 and G-CSF), and proinflammatory (IL-1α, IL-1β, IL-17A, and TNF-α) cytokines and chemokines (CCL3, CCL4, CCL2, CXCL1, and CCL5) were determined. We observed that H99γ immunized mice challenged with *C. neoformans* strain H99 exhibited a significant increase in Th1 associated cytokines (IFN-γ, IL-2, and IL-12p70) as early as day 3 post challenge that remained significantly elevated, except IL-2, through day 7 post challenge compared to HKH99γ immunized mice challenged with H99 (Tables 2 and 3). However, we observed significantly less Th1 associated cytokine (IL-12p70 and IFN-γ) production at day 14 post challenge in H99γ immunized mice compared to HKH99γ immunized mice (Table 4). Also, proinflammatory (IL-1α and IL-1β) and immunoregulatory (G-CSF) cytokines and chemokine levels (CCL3, CCL4, CCL2, and CXCL1) in H99γ immunized mice challenged with H99 were significantly less compared to levels observed in HKH99γ immunized mice challenged with H99 at day 14 post challenge (Table 4). The significant decrease in Th1 associated and proinflammatory cytokine and chemokine levels in H99γ immunized mice challenged with H99 at day 14 post challenge compared to levels observed in HKH99γ immunized mice likely coincides with the significant reduction in pulmonary fungal burden observed in the H99γ immunized mice; whereas, the HKH99γ immunized mice are experiencing progressive disease (Figure 1C). Th1 associated cytokine levels in the lungs of H99γ immunized mice challenged with each other serotype were generally elevated at day 3 post challenge and remained significantly elevated at days 7 and 14 post challenge, particularly cytokines IFN-γ and IL-12p70, compared to their HKH99γ immunized counterparts (Tables 2–4). For Th2-associated cytokines, we observed a significant increase at day 3 post challenge in mice that were immunized with H99γ and challenged with each serotype [H99 (IL-4 and IL-5), R265, WSA87, or R4247 (IL-5)] (Table 2). At day 7 post challenge in mice immunized with H99γ, we observed an increase in IL-4 in mice challenged with R265 or R4247 (Table 3). By day 14 post challenge, there was a statistically significant decrease in Th2 associated cytokines (IL-4 and IL-5) across each serotype except for WSA87 challenged mice that showed significantly increased IL-5 levels across days 7 and 14 post challenge (Tables 3 and 4). We also observed an overall trend toward significantly increased proinflammatory cytokine (IL-1α, IL-1β, and IL-17A), immunoregulatory cytokine (G-CSF), and chemokine (CCL3, CCL4, CCL2, CXCL1, and CCL5) production as early as day 3 post challenge that remained elevated through day 7 post challenge in H99γ immunized mice challenged with each serotype compared to similarly challenged mice immunized using HKH99γ (Tables 2 and 3). We observed an overall trend toward significantly higher levels of proinflammatory cytokine (IL-1α, IL-1β, IL-17A, and TNF-α), immunoregulatory cytokine (G-CSF), and chemokine levels (CCL3, CCL4, CCL2, CXCL1, and RANTES) was observed in H99γ immunized mice challenged with R265, WSA87, or R4247 compared to similarly challenged HKH99γ immunized at day 14 post challenge except for CCL3 and CXCL1 for R265 challenged mice, and CCL3 and CCL2 for R4247 challenged mice (Table 4). Altogether, the cytokine results suggest that immunization with *C. neoformans* strain H99γ elicits a heightened cytokine anamnestic response against multiple disparate serotypes of *Cryptococcus* compared to HKH99γ immunized mice.

**Table 2:** Cytokine levels within lung homogenates of H99γ and HKH99γ immunized mice infected with each serotype.

Day 3 post challenge								

	HKH99γ	H99γ	HKH99γ	H99γ	HKH99γ	H99γ	HKH99γ	H99γ

	H99		R265		WSA87		R4247	
**Th1 associated**								
Interferon (IFN)-γ	5.88 ± 1.45	**12.57** ± 1.84*	4.26 ± 1.07	6.461 ± 0.9474	5.59 ± 1.55	5.94 ± 0.68	3.22 ± 0.71	**6.40** ± 0.89*
IL-2	26.03 ± 6.49	**109.30** ± 18.96**	17.98 ± 3.66	21.3 ± 2.35	22.15 ± 6.41	35.64 ± 4.94	15.09 ± 3.28	**27.58** ± 4.31*
IL-12p70	13.51 ± 0.72	**69.90** ± 8.56***	14.95 ± 0.58	**27.9** ± 1.754***	16.04 ± 4.42	**34.21** ± 3.23**	9.91 ± 1.08	**29.20** ± 3.97***
**Th2 associated**								
IL-4	4.15 ± 1.40	**34.58** ± 6.29***	4.36 ± 1.33	5.873 ± 1.514	4.58 ± 1.31	9.25 ± 2.39	4.22 ± 1.27	6.02 ± 1.42
IL-5	2.49 ± 0.24	**10.96** ± 0.94***	2.26 ± 0.30	**4.729** ± 0.4244***	2.91 ± 0.63	**6.32** ± 0.52***	2.00 ± 0.19	**5.43** ± 0.27***
**Proinflammatory**								
IL-1α	142.40 ± 25.02	**20.86** ± **3.38**^τττ^	16.60 ± 1.85	18.53 ± 0.9751	18.45 ± 3.08	**37.21** ± 4.70**	15.58 ± 1.69	**23.42** ± 2.61*
IL-1β	45.79 ± 4.72	**815.70** ± 124.40***	46.04 ± 6.80	**105.9** ± 8.724***	45.37 ± 2.90	**248.90** ± 40.52***	37.11 ± 1.94	**120.40** ± 18.97***
IL-17A	2.56 ± 0.41	**501.70** ± 83.71***	2.72 ± 0.25	**44.56** ± 8.306***	3.09 ± 0.44	**195.50** ± 43.31***	2.41 ± 0.30	**52.92** ± 12.55**
TNF-α	45.64 ± 5.51	64.35 ± 7.17	40.39 ± 4.26	48.37 ± 3.266	38.08 ± 4.85	**53.17** ± 4.03*	30.81 ± 2.78	**48.19** ± 4.99**
**Immunoregulatory**								
IL-10	5.15 ± 0.61	**23.05** ± 1.96***	4.25 ± 0.76	**11.18** ± 1.105***	4.30 ± 0.51	**12.78** ± 0.81***	3.93 ± 0.55	**8.90** ± 1.09**
G-CSF	5.22 ± 1.25	**510.90** ± 64.82***	5.27 ± 1.19	**45.88** ± 5.85***	5.62 ± 1.30	**135.90** ± 24.62***	4.76 ± 1.21	**64.73** ± 12.34***
**Chemokine**								
CCL3/MIP-1α	43.41 ± 5.17	**452.80** ± 49.90***	45.01 ± 3.49	**111.60** ± 9.96***	68.20 ± 24.17	**194.70** ± 18.11***	37.94 ± 1.68	**122.20** ± 12.11***
CCL4/MIP-1β	6.64 ± 0.81	**79.97** ± 13.09***	6.27 ± 0.53	**16.58** ± 1.97***	6.35 ± 0.70	**35.92** ± 6.91***	4.72 ± 0.21	**17.78** ± 1.94***
CCL2/MCP-1	57.51 ± 8.79	**934.30** ± 88.77***	64.73 ± 4.93	**189.30** ± 21.11***	64.70 ± 6.91	**411.70** ± 95.02**	56.65 ± 6.29	**183.00** ± 17.56***
CXCL1/KC	26.76 ± 1.74	**971.50** ± 94.97***	34.39 ± 4.76	**189.70** ± 31.80***	32.66 ± 2.49	**460.40** ± 79.29***	26.03 ± 1.98	**211.60** ± 31.00***
CCL5/RANTES	152.90 ± 19.47	**1241.00** ± 127.50***	166.60 ± 11.56	**321.00** ± 42.19**	273.70 ± 118.80	**560.70** ± 49.99*	156.00 ± 10.44	**375.60** ± 38.23***

*Data shown are expressed as mean ± SEM and are cumulative of three experiments using four mice per group per time point*.

*Bolded values indicate significance either increased or decreased compared to HKH99γ*.

*p < 0.05, **p < 0.005, ***p < 0.0001 significant increase compared to HKH99γ.

*^^τττ^p < 0.0001 significant decrease compared to HKH99γ^*.

**Table 3:** Cytokine levels within lung homogenates of H99γ and HKH99γ immunized mice infected with each serotype.

Day 7 post challenge								

	HKH99γ	H99γ	HKH99γ	H99γ	HKH99γ	H99γ	HKH99γ	H99γ

	H99		R265		WSA87		R4247	
**Th1 associated**								
IFN-γ	5.23 ± 0.79	**13.28** ± 0.88***	4.83 ± 0.54	**10.36** ± 1.08***	5.29 ± 0.95	**10.40** ± 0.42***	5.70 ± 1.46	**9.61** ± 0.62*
IL-2	19.69 ± 5.12	31.41 ± 3.14	24.52 ± 8.36	35.83 ± 4.22	20.02 ± 4.53	**37.75** ± 5.55*	24.48 ± 6.46	38.61 ± 4.57
IL-12p70	27.22 ± 5.78	**139.60** ± 30.20**	14.19 ± 3.31	**77.97** ± 15.14**	13.12 ± 2.53	**75.36** ± 5.15***	16.58 ± 4.11	**61.84** ± 6.50***
**Th2 associated**								
IL-4	30.90 ± 14.04	12.87 ± 3.31	1.98 ± 1.30	**8.46** ± 1.58**	0.33 ± 0.12	5.19 ± 2.53	0.45 ± 0.16	**4.86** ± 1.29**
IL-5	12.51 ± 2.74	8.41 ± 0.72	8.08 ± 2.36	6.89 ± 0.39	0.96 ± 0.37	**5.73** ± 1.47**	7.05 ± 0.83	6.97 ± 1.16
**Proinflammatory**								
IL-1α	31.91 ± 5.40	**353.40** ± 95.66**	20.30 ± 5.94	**109.40** ± 17.98***	15.39 ± 1.78	**152.30** ± 35.52**	18.42 ± 2.24	**95.36** ± 17.63***
IL-1β	123.10 ± 23.21	**1816.00** ± 409.20**	46.24 ± 4.47	**782.00** ± 137.60***	29.71 ± 3.71	**942.80** ± 206.40***	49.95 ± 7.70	**647.60** ± 121.60***
IL-17A	6.84 ± 2.27	**663.20** ± 90.46***	0.62 ± 0.24	**454.60** ± 83.45***	0.47 ± 0.18	**669.40** ± 210.30**	0.77 ± 0.23	**481.50** ± 112.00***
TNF-α	39.17 ± 3.51	**84.68** ± 5.97***	34.34 ± 1.70	**66.83** ± 3.35***	37.01 ± 3.59	**66.90** ± 3.61***	40.08 ± 4.15	**59.52** ± 3.14**
**Immunoregulatory**								
IL-10	7.31 ± 0.84	**40.08** ± 5.68***	5.30 ± 0.92	**25.05** ± 3.14***	4.02 ± 0.51	**27.08** ± 2.55***	3.87 ± 0.78	**21.09** ± 2.47***
G-CSF	64.74 ± 13.03	**408.90** ± 99.61**	5.92 ± 0.85	**211.10** ± 44.00***	3.22 ± 0.34	**298.50** ± 89.68**	13.12 ± 2.22	**189.40** ± 53.74**
**Chemokine**								
CCL3/MIP-1α	138.00 ± 31.56	**354.40** ± 36.93***	44.37 ± 3.16	**226.90** ± 15.24***	29.94 ± 2.05	**255.40** ± 53.29***	34.75 ± 5.91	**207.70** ± 37.53***
CCL4/MIP-1β	29.95 ± 6.78	**66.32** ± 14.73*	8.82 ± 1.72	**40.40** ± 4.68***	6.86 ± 1.17	**43.55** ± 6.54***	6.77 ± 1.42	**31.96** ± 3.67***
CCL2/MCP-1	372.60 ± 95.97	**962.50** ± 152.70**	89.06 ± 14.96	**713.00** ± 127.20***	38.46 ± 5.58	**636.00** ± 64.17***	116.90 ± 28.72	**474.90** ± 58.73***
CXCL1/KC	268.20 ± 47.52	**1055.00** ± 169.70***	58.81 ± 7.29	**774.70** ± 118.20***	45.73 ± 14.61	**782.60** ± 140.60***	86.95 ± 15.98	**643.10** ± 111.40***
CCL5/RANTES	246.70 ± 31.91	**1407.00** ± 156.60***	135.20 ± 6.51	**621.90** ± 59.09***	105.80 ± 8.11	**880.10** ± 183.90***	86.45 ± 11.71	**607.50** ± 110.20***

*Data shown are expressed as mean ± SEM and are cumulative of three experiments using four mice per group per time point*.

*Bolded values indicate significance either increased or decreased compared to HKH99γ*.

*p < 0.05, **p < 0.005, ***p < 0.0001 significant increase compared to HKH99γ

**Table 4:** Cytokine levels within lung homogenates of H99γ and HKH99γ immunized mice infected with each serotype.

Day 14 post challenge								

	HKH99γ	H99γ	HKH99γ	H99γ	HKH99γ	H99γ	HKH99γ	H99γ

	H99		R265		WSA87		R4247	
**Th1 associated**								
IFN-γ	8.37 ± 0.60	**6.33** ± **0.54**^τ^	7.09 ± 0.74	**11.71** ± 0.88**	4.61 ± 0.57	**10.52** ± 0.66***	4.80 ± 0.23	**10.82** ± 0.94***
IL-2	15.67 ± 1.19	15.87 ± 3.17	14.14 ± 0.43	**28.83** ± 2.17***	13.21 ± 4.47	**28.20** ± 1.48**	9.67 ± 1.21	**28.49** ± 1.09***
IL-12p70	78.16 ± 10.72	**30.11** ± **2.46**^τττ^	62.74 ± 9.46	**130.30** ± 23.33*	21.28 ± 2.23	**127.10** ± 20.38***	41.41 ± 3.31	**135.10** ± 21.07***
**Th2 associated**								
IL-4	190.30 ± 33.23	**1.37** ± **0.37**^τττ^	102.40 ± 17.90	**14.11** ± **4.43**^τττ^	6.38 ± 2.57	4.96 ± 0.96	68.98 ± 7.51	**12.23** ± **2.89**^τττ^
IL-5	23.74 ± 5.57	**1.83** ± **0.70**^ττ^	73.75 ± 21.66	**8.24** ± **0.98**^ττ^	4.95 ± 1.27	**8.88** ± 1.09*	74.35 ± 13.94	**7.35** ± **0.86**^τττ^
**Proinflammatory**								
IL-1α	151.60 ± 18.96	**20.36** ± **2.50**^τττ^	122.20 ± 12.17	**380.80** ± 84.73**	29.50 ± 6.28	**337.90** ± 46.85***	105.90 ± 14.39	**471.00** ± 39.58***
IL-1β	862.80 ± 218.50	**87.04** ± **8.71**^ττ^	398.20 ± 71.61	**2434.00** ± 519.10**	82.79 ± 21.65	**2346.00** ± 383.20***	315.20 ± 53.03	**2645.00** ± 322.50***
IL-17A	55.84 ± 10.26	33.87 ± 6.83	17.10 ± 4.86	**959.40** ± 178.60***	3.24 ± 1.27	**897.10** ± 105.60***	6.65 ± 2.03	**968.90** ± 83.29***
TNF-α	52.10 ± 2.22	46.05 ± 3.00	45.81 ± 2.05	**80.03** ± 6.04***	32.51 ± 2.89	**76.46** ± 4.20***	38.77 ± 1.57	**71.21** ± 4.29***
**Immunoregulatory**								
IL-10	26.14 ± 1.78	**11.61** ± **0.93**^τττ^	27.59 ± 3.00	**40.29** ± 4.54*	7.96 ± 0.98	**39.11** ± 3.23***	17.97 ± 1.63	**40.08** ± 3.30***
G-CSF	62.52 ± 15.47	**5.02** ± **0.42**^ττ^	42.48 ± 4.68	**81.41** ± 13.82*	13.80 ± 2.37	**110.50** ± 20.14***	35.52 ± 4.65	**88.34** ± 10.39***
**Chemokine**								
CCL3/MIP-1α	1023.00 ± 252.10	**99.74** ± **21.77**^ττ^	515.30 ± 77.06	362.80 ± 47.70	73.85 ± 15.04	**346.10** ± 41.46***	286.90 ± 44.40	366.10 ± 23.44
CCL4/MIP-1β	40.83 ± 8.61	**8.71** ± **1.24**^ττ^	21.83 ± 4.15	**40.93** ± 4.74**	8.96 ± 2.82	**36.65** ± 4.08***	18.59 ± 1.98	**40.43** ± 2.62***
CCL2/MCP-1	542.50 ± 117.10	**93.71** ± **11.92**^ττ^	1244.00 ± 213.40	**516.20** ± **71.17**^ττ^	183.80 ± 51.99	**585.40** ± 61.80***	827.00 ± 188.20	486.40 ± 35.50
CXCL1/KC	533.10 ± 57.85	**80.39** ± **10.84**^τττ^	496.70 ± 65.65	655.90 ± 70.33	297.00 ± 67.81	**751.40** ± 77.80***	345.20 ± 55.87	**625.50** ± 31.55***
CCL5/RANTES	582.60 ± 114.80	305.40 ± 71.84	285.20 ± 27.58	**1402.00** ± 240.60***	201.50 ± 39.46	**1623.00** ± 219.30***	214.70 ± 45.55	**1377.00** ± 162.90***

*Data shown are expressed as mean ± SEM and are cumulative of three experiments using four mice per group per time point*.

*Bolded values indicate significance either increased or decreased compared to HKH99γ*.

*p < 0.05, **p < 0.005, ***p < 0.0001 significant increase compared to HKH99γ.

*^τ^p < 0.05, ^ττ^p < 0.005, ^τττ^p < 0.0001 significant decrease compared to HKH99γ*.

### Vaccine-Mediated Protection against Pulmonary Cryptococcosis Is Associated with Enhanced Th1-Type Immune Responses

**Figure 3:**
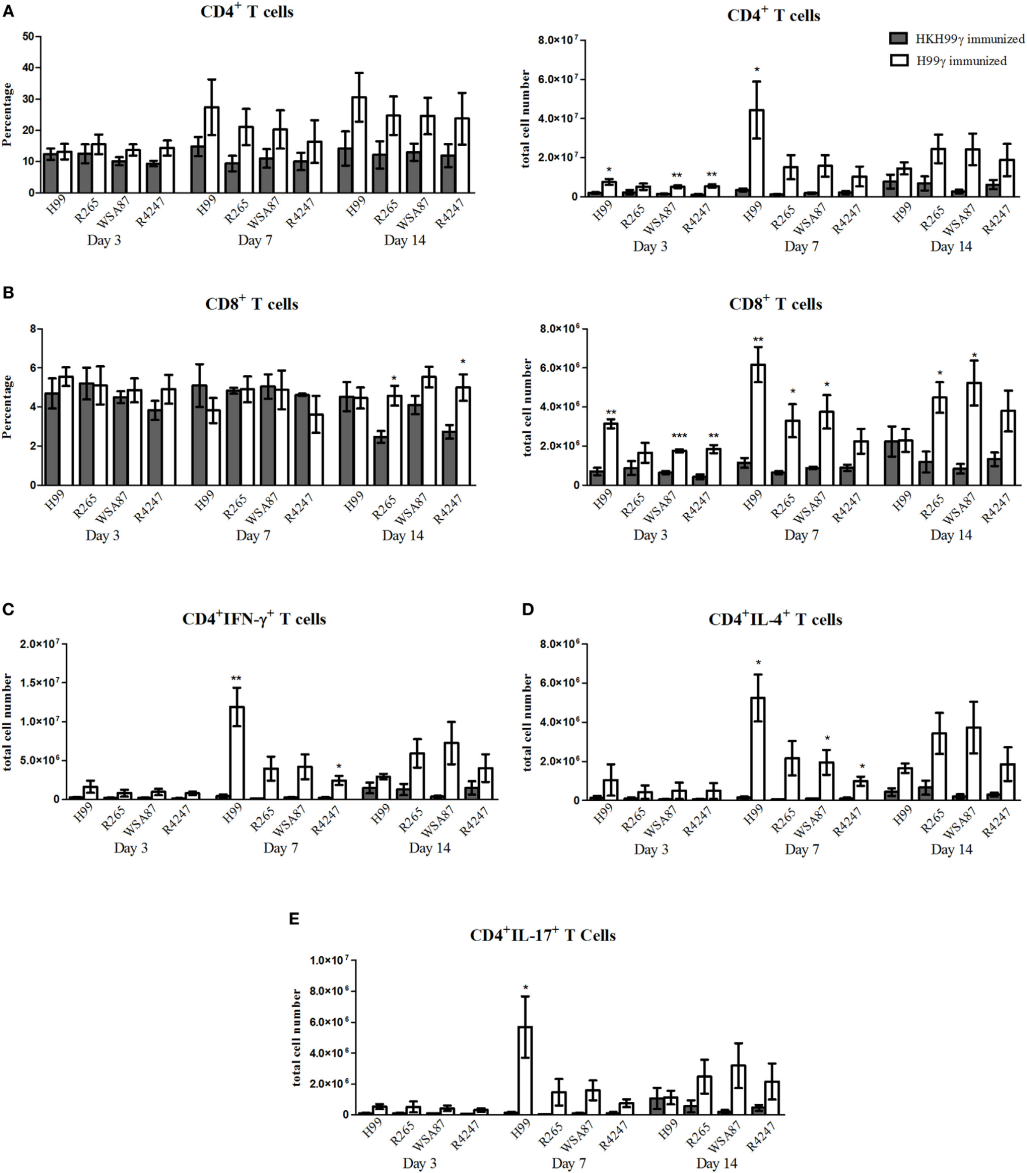
Pulmonary T cell recruitment in immunized mice challenged with disparate serotypes of *Cryptococcus*. BALB/c mice were immunized intranasally with 1 × 10^4^ CFU of either H99γ or HKH99γ, allowed 70 days to resolve the infection, and subsequently challenged with one serotype of *Cryptococcus*. At days 3, 7, and 14 post challenge, lungs were excised, tissues digested, and pulmonary immune cell infiltrates were analyzed by flow cytometry **(A,B)** as described previously in Figure 2 or further permeabilized to stain for intracellular cytokines **(C–E)**. Data shown are the mean ± SEM of absolute cell numbers or percentage from three independent experiments performed using 4 mice per group per experiment. *p < 0.05, **p < 0.005, ***p < 0.0001. *Significant increase compared to HKH99γ.

We next evaluated T cell responses in the lungs of immunized mice following challenge with the various *Cryptococcus* serotypes. Flow cytometry analysis was utilized to determine levels of CD4^+^ and CD8^+^ T cell infiltration to the lungs on days 3, 7, and 14 post challenge. Also, intracellular cytokine staining of CD4^+^ T cells for representative T helper (Th) 1, Th2, and Th17-type cytokines IFN-γ, IL-4, and IL-17A, respectively, and subsequent flow cytometry analysis was used to access the CD4^+^ Th cell phenotype during the anamnestic response to pulmonary cryptococcosis. We observed an overall trend toward increased total numbers of CD4^+^ and CD8^+^ T cells in H99γ immunized mice compared to HKH99γ immunized mice following challenge with each serotype as the infection progressed (Figures 3A,B). The total number of CD4^+^ T cells was significantly increased in H99γ immunized mice challenged with H99, R265, or R4247 at day 3 post challenge, and in mice challenged with H99 at day 7 post challenge compared to their HKH99γ immunized counterparts (Figure 3A). However, there was no significant difference in percentage of CD4^+^T cells between groups. We also observed an increase in the total number of CD8^+^ T cells in H99γ immunized mice challenged with H99, WSA87, or R4247 at day 3 post challenge, in mice challenged with H99, R265, or WSA87 at day 7 post challenge, and in R265 or WSA87 challenged mice at day 14 post challenge compared to HKH99γ immunized mice challenged with their equivalent serotype (Figure 3B). A significant increase in the percentage of CD8^+^ T cells was observed in mice immunized with H99γ and challenged with R265 or R4247 at day 14 post challenge compared to their HKH99γ immunized counterparts.

Overall, we observed significant increases in the total number of CD4^+^/IFN-γ^+^ T cells followed by CD4^+^/IL-17A^+^ T cells and then CD4^+^/IL-4^+^ T cells at day 7 post challenge in H99γ immunized mice challenged with each serotype compared to HKH99γ immunized mice challenged with the corresponding serotype (Figures 3C–E). We observed greater total CD4^+^/IFN-γ^+^ T cell numbers in H99γ immunized mice challenged with H99 compared to H99γ immunized mice challenged with R265, WSA87, or R4247 at day 7 post challenge (Figure 3C). These results suggest that mice immunized with H99γ are capable of eliciting a predominantly Th1-type anamnestic response upon challenge with each disparate *Cryptococcus* serotype. These data show that mice immunized with the serotype A strain exhibit an increased putatively protective Th1-type immune response following challenge with other disparate serotypes.

### Protection Afforded by Immunization with H99γ against Serotypes B and D of *Cryptococcus* Requires CD4^+^ T Cells

**Figure 4:**
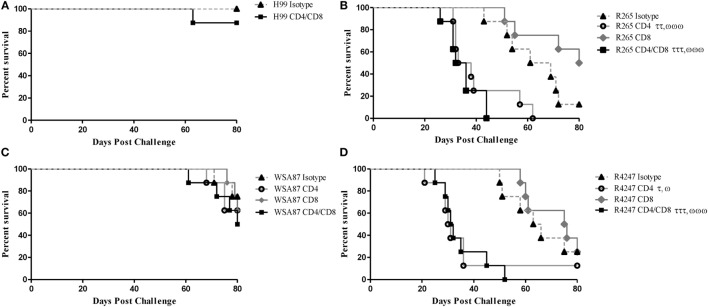
Protection mediated by immunization with *C. neoformans* strain H99γ, serotype A, appears to be predominantly CD4^+^ T cell-mediated. BALB/c mice were immunized intranasally with 1 × 10^4^ CFU of H99γ and allowed 70 days to rest. Mice were depleted of CD4^+^ T cells, CD8^+^ T cells, or both CD4^+^/CD8^+^ T cell subsets or received isotype control antibody two days prior to challenge with one serotype of *Cryptococcus* and weekly thereafter during the observation period. Mice were observed for up to 80 d post challenge for survival analysis. Survival data shown are from one experiment each, using 8 mice per group. However, the results are shown for individual groups and mice challenged with H99 **(A)**, R265 **(B)**, WSA87 **(C)** or R4247 **(D)** for simplicity. ^τ^*p* < 0.05, ττ*p* < 0.005, ^τττ^*p* < 0.0001. τ = significant decrease compared to isotype; ^ω^*p* < 0.05, ^ωω^*p* < 0.005, ωωω*p* < 0.0001. ω = significant decrease compared to CD8^+^ T cell depleted.

Previous studies in our lab demonstrated that H99γ immunized mice depleted of CD4^+^ or CD8^+^ T cells were completely protected against an otherwise lethal challenge with the non-IFN-γ producing *C. neoformans* strain H99 (40). Also, H99γ immunized mice that were subsequently depleted of both CD4^+^ and CD8^+^ T cells were shown to have an 80% survival rate following challenge with *C. neoformans* strain H99. Therefore, we sought to determine the necessity of T cells in protection against cryptococcosis caused by the different serotypes of *Cryptococcus* in H99γ immunized mice. For this, BALB/c mice were immunized with *C. neoformans* strain H99γ and allowed 70 days to resolve the infection. Mice were then depleted of CD4^+^ and/or CD8^+^ T cells or received isotype control antibody two days prior to challenge and weekly thereafter during the observation period. H99γ immunized mice depleted of both CD4^+^ and CD8^+^ T cells prior to and during challenge with H99 demonstrated an 87.5% survival rate upon the conclusion of the study (Figure 4A) as previously demonstrated (40). Figure 4 shows that H99γ immunized mice treated with isotype control antibodies and challenged with R265 (Figure 4B) or R4247 (Figure 4D) had similar prolonged survival (median survival times of 65 and 64.5 days for R265 and R4247 challenged mice, respectively); similar to observations demonstrated in Figure 1B. In contrast, we observed 100% mortality in H99γ immunized mice depleted of CD4^+^ T cells alone during challenge with R265 (median survival of 35.5 days) or depleted of CD4^+^ and CD8^+^ T cells (median survival of 34 days; Figure 4B). Interestingly, H99γ immunized mice depleted of CD8^+^ T cells alone and challenged with R265 showed increased, although not statistically significant, survival (median survival of 80 days) compared to that observed in isotype control-treated mice (median survival of 65 days). We also observed that H99γ immunized mice challenged with R4247 while depleted of CD4^+^ T cells alone experienced 87.5% mortality (median survival time of 30.5 days) and 100% mortality upon depletion of both CD4^+^ and CD8^+^ T cells (median survival time of 31.5 days; Figure 4D). Additionally, H99γ immunized mice depleted of CD8^+^ T cells alone and challenged with R4247 had increased survival although not statistically significant (median survival of 75.5 days) compared to the isotype control-treated mice (median survival of 64.5 days). Mice immunized with H99γ and subsequently depleted of CD4^+^ T and/or CD8^+^ T cells prior to and during challenge with WSA87 did not show any difference in survival compared to the isotype control-treated mice (Figure 4C). Overall, we observed that depletion of CD4^+^ T cells resulted in a significant loss of protection against challenge with R265 (serotype B) or R4247 (serotype D) in H99γ (serotype A) immunized mice, indicating that CD4^+^ T cells are required for broad-spectrum protection against these serotypes.

## Discussion

While the overwhelming majority of *Cryptococcus* exposures do not progress to life-threatening illness, the ubiquitous presence of *Cryptococcus* in the environment world-wide indicates that exposure to persons predicted to be at an exceptionally high risk for developing cryptococcosis (i.e., patients scheduled to receive organ transplants, otherwise healthy HIV^+^ persons, and immune competent persons in areas observed to contain *C. gattii*) is inevitable and supports the need for development of a prophylactic and/or therapeutic vaccine that provides broad-spectrum protection against multiple *Cryptococcus* serotypes (9–16, 42). We have previously shown that mice immunized with *C. neoformans* strain H99γ, serotype A, are protected against a subsequent otherwise lethal challenge with wild-type *C. neoformans* strain H99 (25, 36–38, 40, 41, 43–45). Herein, we demonstrate the potential for achieving broad-spectrum protection against multiple clinically relevant strains of *Cryptococcus* serotypes following vaccination with a *Cryptococcus* serotype A strain previously shown to elicit protective immunity against *C. neoformans*. Specifically, we demonstrated that mice immunized with *Cryptococcus* strain H99γ, serotype A, develop significant protection against challenge with serotypes A, B, or D of *Cryptococcus*. Our data are in line with a similar study showing that mice vaccinated with an avirulent chitosan-deficient *C. neoformans* strain also created from a serotype A background strain exhibited significantly delayed mortality when challenged with serotype B *C. gattii* strains R265 or WM276 (34). Altogether, these studies clearly demonstrate that significant cross-protection can be developed following vaccination with a *Cryptococcus* strain of a disparate serotype and provide further proof-of-principle for the development of a prophylactic vaccine that can protect against multiple disparate *Cryptococcus* serotypes.

Both clinical and experimental studies have shown that Th1-type CD4^+^ T CMI is critical for protection against cryptococcosis (24, 25, 46). We observed a predominantly Th1-type and proinflammatory cytokine profile and increased survival in H99γ immunized mice following challenge with representative *Cryptococcus* serotypes B or D. Additionally, intracellular cytokine staining of CD4^+^ T cells showed that H99γ immunized mice were capable of eliciting a predominantly Th1-type anamnestic immune response upon subsequent challenge with the other *Cryptococcus* serotypes. These studies indicated that a Th1-type cell-mediated immune response was responsible for the increased survival observed in H99γ immunized mice challenged with *Cryptococcus* strains from serotypes B or D. We also observed increases in some Th2-type cytokines in H99γ immunized mice; however, the overall low levels observed are unlikely to be biologically relevant and were not as significant as the levels observed in HKH99γ immunized mice at day 14 post challenge. These findings do suggest that a Th1-Th2 balance may be important to achieve an optimal host immune response against *Cryptococcus* (47). Additionally, we observed an overall increase in the total number of infiltrating leukocytes in H99γ immunized mice compared to HKH99γ immunized mice challenged with their equivalent serotypes. Dendritic cells are known to play a vital role in the initial response against *C. neoformans* with the ability to phagocytose and kill the fungal organisms (48, 49). The significant increase in the infiltration of CD11b^+^/CD11c^+^ cells in mice given the H99γ immunization prior to challenge with the different serotypes compared to HKH99γ immunized mice correlates with reduced fungal burden in these mice. We did observe an increase in F4/80^+^ cells in H99γ immunized mice. However, previous studies in our laboratory has demonstrated that quantity of macrophages does not correlate with clearance, rather the ability of F4/80^+^ cells to polarize to a classically activated, M1 phenotype promotes clearance of *C. neoformans* (36, 41, 43, 50). The increase in infiltrating Ly6G^+^ cells correlates with significant increases in known neutrophil chemoattractants [IL-17A, G-CSF (indirect chemotaxis), and CXCL1/KC] in the H99γ immunized mice (51, 52). However, previous studies from our lab demonstrate that neutrophils are not critical for clearance of *C. neoformans* strain H99 in H99γ immunized mice (44).

The vast majority of patients who acquire cryptococcosis due to *C. neoformans* are severely immunocompromized while those who acquire *C. gattii* infections appear to have little to no known immunodeficiency (53–55). Nonetheless, the protection observed in H99γ immunized mice challenged with serotypes B or D appeared to be CD4^+^ T cell-dependent. Interestingly, depletion of CD8^+^ T cells appeared to partially enhance survival in H99γ immunized mice challenged with serotypes B or D. We hypothesize that this additional protection may be attributed to a reduction in putative deleterious CD8^+^ T cell-mediated inflammatory responses or regulatory function in CD8^+^ T cell depleted mice challenged with serotypes B or D; however, this hypothesis will need to be confirmed in follow-up studies. In contrast, significant protection was evident in H99γ immunized mice rendered CD4^+^ and CD8^+^ T cell deficient and challenged with the serotype A *Cryptococcus* strain, H99. These studies demonstrate that immunization with H99γ produces memory T cells that allow for a rapid response against the invading cryptococcal strain as previously shown in our studies (40). Subtle species-specific differences in an antigen’s peptide sequence and/or structure in the different serotypes tested may account for the lack of complete protection observed in our studies. Without the memory T cells present, there is no delay in onset of disease and the CD4^+^ T cell deficient mice challenged with serotypes B or D succumb to infection at a rate similar to the mice that were immunized with HKH99γ. Recent studies have shown increased protection against *C. neoformans* and *C. gattii* using a vaccine formulation comprised of antigens extracted from capsule or chitosan deficient *Cryptococcus* strains by treatment with an alkaline solution and packaged into GPs (33). The source of antigens for each vaccine formulation was specifically tailored to each challenge (i.e., *C. neoformans* antigens for *C. neoformans* challenge), and thus, the efficacy for the formulations to provide cross-protection was not determined. However, these studies also suggested that the protection induced following immunization with the *Cryptococcus* antigen/GP formulation against *C. neoformans* challenge was dependent on T CMI (33). The protection observed in T cell-deficient, H99γ immunized mice against a subsequent serotype A challenge may be antibody-mediated or may be suggestive of a trained innate cell population that compensates for the lack of T cells. Although we did observe an increase in the total number of B cells in H99γ immunized mice compared to HKH99γ immunized mice on days 7 and 14 post challenge, previous studies from our lab show that B cells are not required for the generation of protective immunity against cryptococcosis in H99γ immunized mice suggesting that antibody-mediated protection in the absence of T cells is unlikely (25). Overall, immunization with H99γ resulted in a non-T cell dependent protective immune response to challenge with a serotype A *Cryptococcus* strain showing that long-term protection in immunocompromized hosts can be generated. However, our results showing that CD4^+^ T cells are required for the protection observed in H99γ immunized mice challenged with serotypes B and D suggests that a vaccine formulation designed to induce complete protection against multiple *Cryptococcus* serotypes should include antigens specific to each serotype or contain antigens with significant homology across the serotypes.

Our studies also showed that the serotype C *Cryptococcus* strain used herein, WSA87, is less virulent or remains latent in mice compared to all the other strains tested. When H99γ immunized mice were subsequently challenged with WSA87, we observed a statistically significant increase in immune cell infiltrates at day 7 post challenge compared to HKH99γ immunized mice which correlated with the significant decrease of fungal burden in H99γ immunized mice. However, survival during challenge with serotype C was similar regardless of the immunization strategy most likely due to the initial low virulence of this strain or the strain’s ability to remain latent with mice not succumbing to infection until almost day 90 post challenge. Also, depletion of CD4^+^ and/or CD8^+^ T cells was observed to have no impact on the survival of H99γ immunized mice challenged with WSA87. The results suggest that WSA87 suppresses T CMI or that protection against WSA87 is not dependent on T CMI. The serotype C strain chosen for this study was a clinical isolate that caused human disease; however, virulence due to this strain appeared to be relatively low in mice. *Cryptococcus* strains of the same serotype are known to exhibit significant variability in virulence within murine models. Thus, it is inappropriate to suggest that the serotypes utilized in our study are completely representative of all *Cryptococcus* strains within their serotype. However, these specific results suggest an unfortunate and perhaps unavoidable reality that developing vaccine formulations that target the more pathogenic *Cryptococcus* strains may still allow for less virulent strains of *Cryptococcus* to slip under the host’s immunological radar resulting in disease and mortality.

While no fungal vaccines are clinically available, an ideal vaccine would mirror the results of the currently available vaccines against other microbial pathogens that can protect against multiple serotypes. The quadrivalent meningococcal conjugate vaccine (MenACWY-D; Menactra^®^, Sanofi Pasteur, Swiftwater, PA, USA), for example, contains polysaccharides from serotypes A, C, Y, and W-135 meningococci conjugated to a diphtheria protein carrier (56) and provides broad-spectrum protection against those serotypes that cause the majority of meningococcal disease. While the studies presented herein determined the efficacy of vaccination with one serotype to provide cross-protection against other serotypes, our results suggest that the identification and inclusion of multiple cross-protective CD4^+^ T cell-dependent antigens will be critical for the development of a vaccine formulation that elicits broad-spectrum protection against *Cryptococcus* strains that cause the majority of clinical disease. Although much work remains toward the development of a cross-protective cryptococcal vaccine, these studies provide a foundation for understanding the protective host immune response to multiple clinically relevant strains of *Cryptococcus* and the need to identify protective CD4^+^ T cell epitopes that can be incorporated into a vaccine formulation that provides broad-spectrum protection against cryptococcosis.

## Ethics Statement

This study was carried out in accordance with the recommendations of the Institutional Animal Care and Use Committee (protocol number MU021) of the University of Texas at San Antonio.

## Author Contributions

FW designed study, performed statistical analysis, interpreted study results, and participated in drafting and editing of manuscript. MD assisted in study design, performed experiments and statistical analysis, participated in interpretation of results, and drafted manuscript. AC, SH, CW, NC-L, CH, and KW performed experiments and statistical analysis, participated in interpretation of results and manuscript revision.

## Conflict of Interest Statement

The authors declare that the research was conducted in the absence of any commercial or financial relationships that could be construed as a potential conflict of interest.
