# Supplementary figures and images for: Induction of Broad-Spectrum Protective Immunity against Disparate Cryptococcus Serotypes
Source: Front Immunol. 2017 Oct 30;8:1359. doi: 10.3389/fimmu.2017.01359 (PMC5670106; doi:10.3389/fimmu.2017.01359)

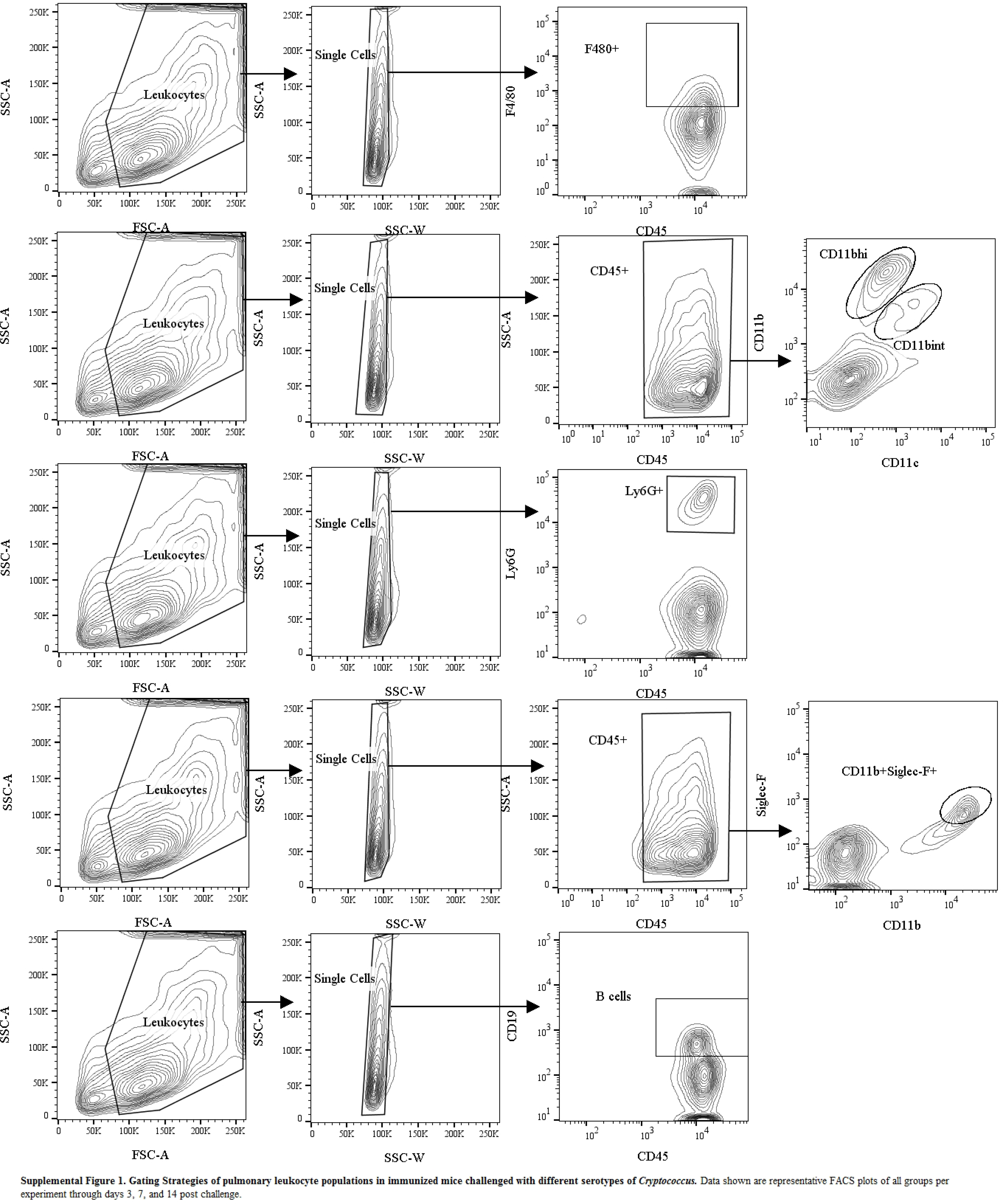

Supplement: Supplementary file 1 [file Image_1.TIF]

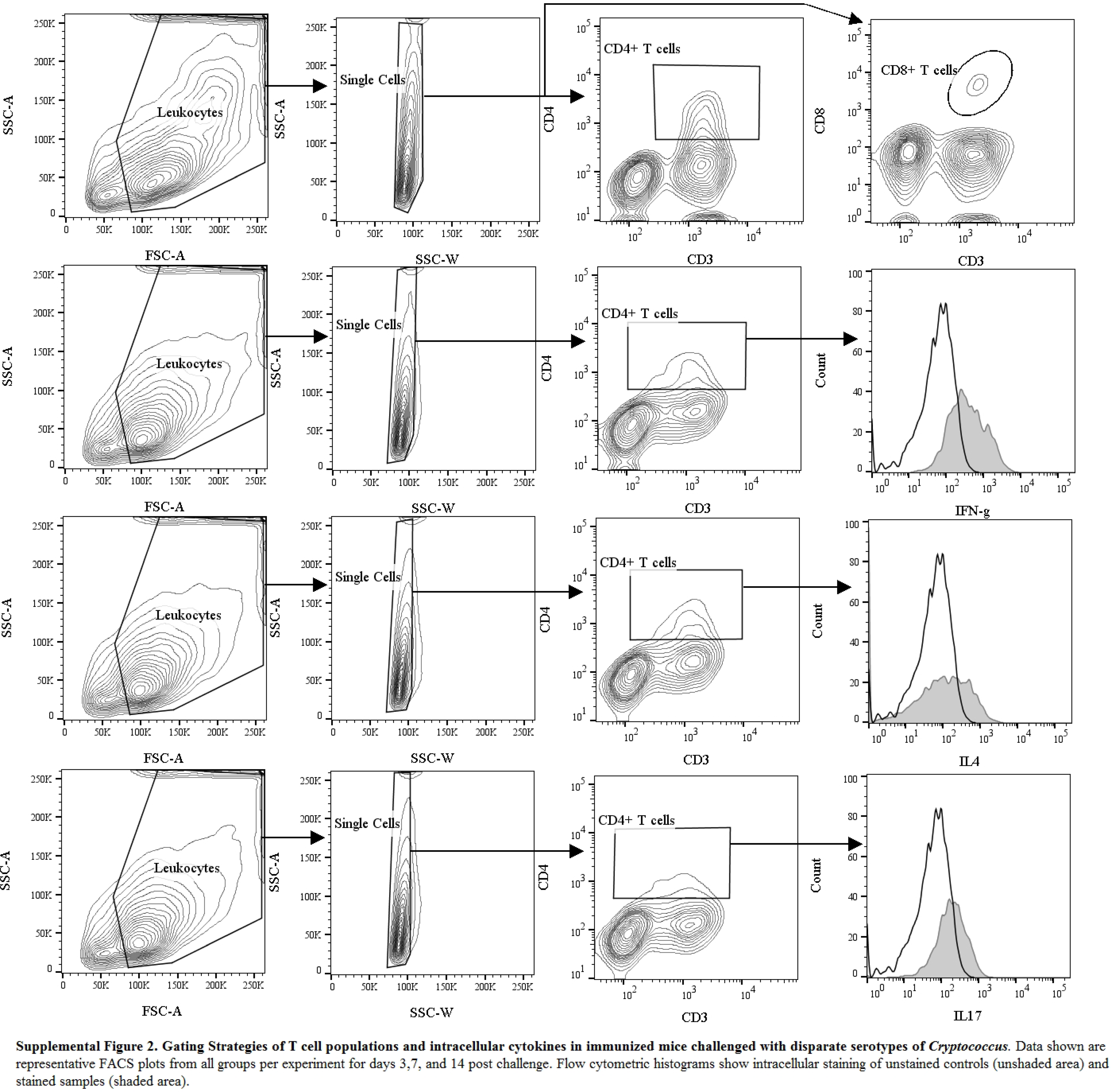

Supplement: Supplementary file 2 [file Image_2.TIF]
